# Influence of Aberration-Free, Narrowband Light on the Choroidal Thickness and Eye Length

**DOI:** 10.1167/tvst.13.4.30

**Published:** 2024-04-25

**Authors:** Susanna P. Clement, Katharina Breher, Niklas Domdei, Josefine Dolata, Siegfried Wahl

**Affiliations:** 1 Institute for Ophthalmic Research, University of Tübingen, Tübingen, Germany; 2 Carl Zeiss Vision International GmbH, Aalen, Germany; 3 Ernst Abbe Hochschule Jena, Jena, Germany

**Keywords:** axial length, choroidal thickness, longitudinal chromatic aberration, narrowband light, broadband light

## Abstract

**Purpose:**

To determine whether light chromaticity without defocus induced by longitudinal chromatic aberration (LCA) is sufficient to regulate eye growth.

**Methods:**

An interferometric setup based on a spatial light modulator was used to illuminate the dominant eyes of 23 participants for 30 minutes with three aberration-free stimulation conditions: (1) short wavelength (450 nm), (2) long wavelength (638 nm), and (3) broadband light (450–700 nm), covering a retinal area of 12°. The non-dominant eye was occluded and remained as the control eye. Axial length and choroidal thickness were measured before and after the illumination period.

**Results:**

Axial length increased significantly from baseline for short-wavelength (*P* < 0.01, 7.4 ± 2.2 µm) and long-wavelength (*P* = 0.01, 4.8 ± 1.7 µm) light. The broadband condition also showed an increase in axial length with no significance (*P* = 0.08, 5.1 ± 3.5 µm). The choroidal thickness significantly decreased in the case of long-wavelength light (*P* < 0.01, −5.7 ± 2.2 µm), but there was no significant change after short-wavelength and broadband illumination. The axial length and choroidal thickness did not differ significantly between the test and control eyes or between the illumination conditions (all *P* > 0.05). Also, the illuminated versus non-illuminated choroidal zone did not show a significant difference (all *P* > 0.05).

**Conclusions:**

All stimulation conditions with short- and long-wavelength light and broadband light led to axial elongation and choroidal thinning. Therefore, light chromaticity without defocus induced by LCA is suggested to be insufficient to regulate eye growth.

**Translational Relevance:**

This study helps in understanding if light chromaticity alone is a sufficient regulator of eye growth.

## Introduction

Research conducted on animal and human models suggests that the choroid can play a vital role in myopia development and progression, as rapid elongation of eye length has been found to be associated with thinning of the choroid and shortening of axial length with choroidal thickening.^1^^–^^3^ In the search for therapies to stop or slow down myopia progression, it has been observed that axial length and choroidal thickness change depending on the light spectrum used. On one hand, a decrease in axial length was noted when guinea pigs and chicks were raised in short-wavelength light (center wavelength of 430 nm),^4^^,^^5^ and when they were raised in long-wavelength (center wavelength of 620 nm) or mid-wavelength light (center wavelength of 530 nm) conditions, an increase in axial length was noted.^5^^–^^7^ On the other hand, tree shrews and rhesus monkeys reared in longer wavelength light showed slower eye growth and a thickening of the choroid.^8^^,^^9^ Studies on emmetropic and myopic humans revealed that exposing the eye for 1 hour to long-wavelength light, darkness, and broadband light resulted in choroidal thinning and an increase in axial length compared to short-wavelength light.^10^^,^^11^ In contrast, clinical studies on myopic children treated with a low-power, long-wavelength laser twice daily for 3 minutes saw a significant reduction in axial length and an increase in choroidal thickness.^12^^,^^13^ Interestingly, in a study done by Thakur et al.,^11^ a significant decrease in axial length was observed for short-wavelength light even in the presence of a hyperopic defocus. The authors concluded that chromatic signals alone may not be enough to regulate eye growth, making it unclear if the eye is reacting to the chromaticity of light or to spectrum-dependent aberrations, such as defocus caused by the longitudinal chromatic aberration (LCA)^14^. LCA is a phenomenon in which the shorter wavelengths are refracted more and focus in front of the retina, causing a myopic defocus, whereas the longer wavelengths are refracted less and focus behind the retina, causing a hyperopic defocus.^15^

With respect to the contradictory study results, the mechanism of light processing as a regulator of eye growth has not yet been clarified, suggesting that the LCA can either be used as a target, directing axial length growth to align the retina with the respective focus of the dominant wavelength, or as a cue, where the dominant wavelength indicates the growth direction to achieve emmetropization.^15^

In previous studies that used commercial lighting devices, the inherent association between light chromaticity (wavelength of light) and LCA-induced defocus could not be determined. In order to separate the two factors of chromaticity and LCA-induced defocus, a pilot study examined the effects of short-, long-, and mid-wavelength light while controlling for the LCA on axial length.^16^ There were no significant changes observed in the axial length for any of the three conditions. This led to an assumption that the response of eyes in previous studies was mainly influenced by the LCA-induced defocus rather than the chromaticity. However, despite the absence of significant changes, axial length has shown a slight tendency to increase for long- and mid-wavelength light, whereas short-wavelength light exposure has shown a tendency for decreased axial length.^16^ In the study by Breher et al.,^16^ this association may not have been very evident due to the relatively small intensity of 4 trolands (Td) and limited stimulation time.

Based on these findings, the aim of the current study was to further explore the changes in axial length and choroidal thickness under the influence of aberration-free narrowband light conditions by using increased light intensities and stimulation time.

## Methods

### Participant Data

A prospective study was conducted at the University of Tübingen, Germany. The participant's data were pseudonymized to maintain data security. The research protocol followed the tenets of the Declaration of Helsinki and was approved by the University of Tübingen medical committee. The inclusion criteria were a spherical refractive error of ≤−6.00 diopters (D), a best-corrected visual acuity of at least 0.1 logMAR, normal color vision, and no self-reported ocular pathologies. Because personalized bite bars were utilized to fix the participant's head during the main experimental procedure, participants with back and neck problems were excluded from the study, as well. In total, 23 participants (15 female) were enrolled in the study, with a mean age of 25 ± 4 years. Objective (ZEISS i.Profiler^plus^; Carl Zeiss Vision International GmbH, Aalen, Germany) and subjective refractions, as well as best-corrected visual acuity (ZEISS VISUPHOR 500 and VISUSCREEN 500), were determined prior to the main measurement. The mean spherical equivalence values for the dominant eyes and the non-dominant eyes were −1.29 ± 1.67 D and −1.14 ± 1.40 D, respectively, and the mean visual acuity values were −0.05 ± 0.06 logMAR and −0.06 ± 0.07 logMAR. Color vision was tested using the Ishihara Test (OCULUS Optikgeräte GmbH, Wetzlar, Germany).

### Aberration-Free Optical Illumination Setup

**Figure 1.**
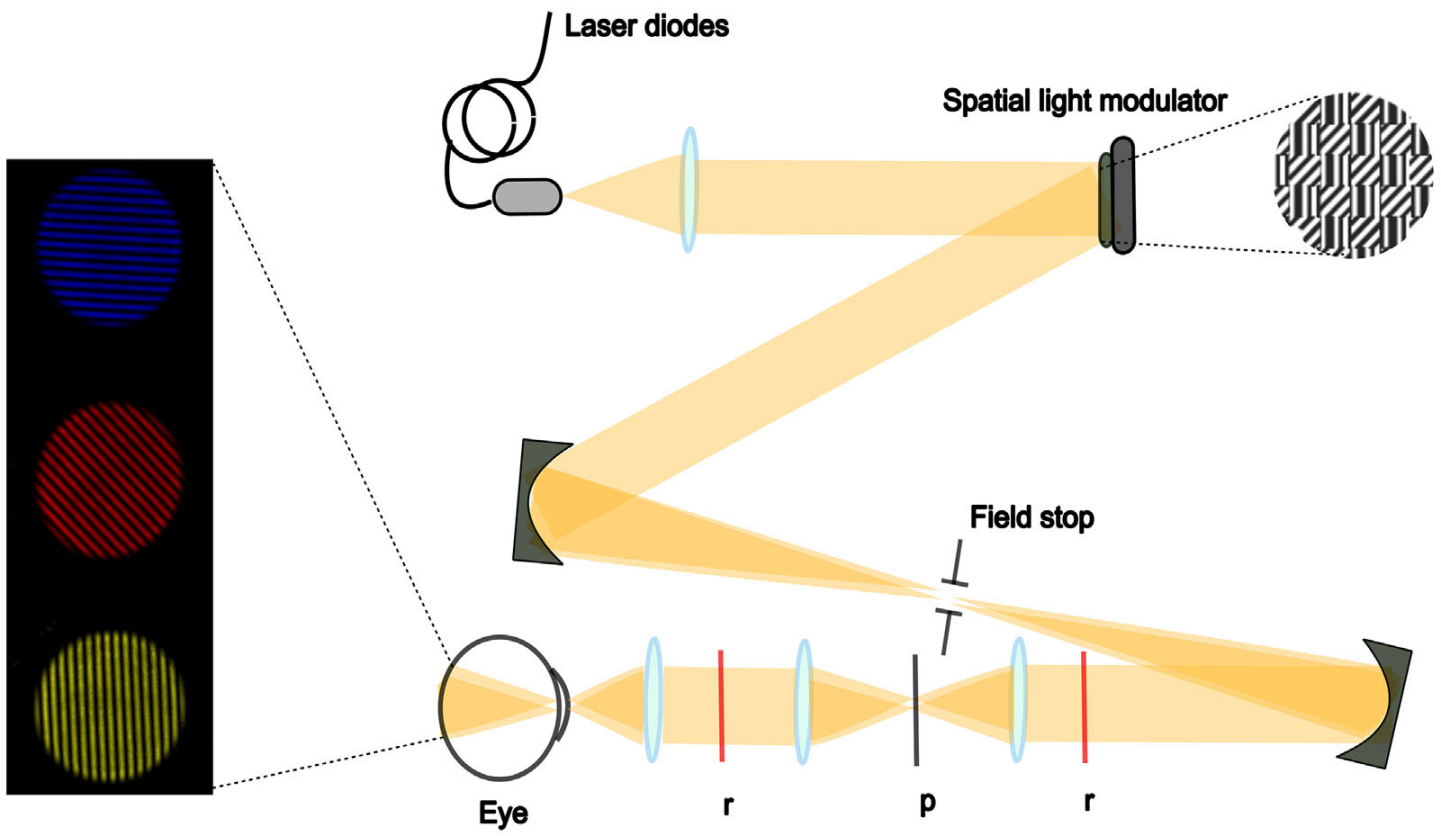
Schematic representation of aberration-free retinal projection by the interferometric setup based on a SLM (adapted from Breher et al.^16^). The *red lines* (r) represent the conjugate plane of the SLM and retina, and the *black line* (p) represents the pupil plane. The *left part* of the image shows the aberration-free stimulus for short-wavelength, long-wavelength, and broadband light.

An interferometric setup based on a spatial light modulator (SLM) (Fig. 1, right) was used to illuminate the eye.^17^ The system uses a Maxwellian view configuration^18^, where two laterally separated wavefronts, generated by the SLM, are focused on the pupil plane of the eye, diverge beyond, and form interference fringes on the retina.^17^ Thereby the system bypasses the optics of the eye and allows to project aberration-free narrowband or polychromatic light stimuli onto the retina. A 12° field of view was achieved by using a lens with a 40-mm focal length (AC254-040-A-ML; Thorlabs, Bergkirchen, Germany) to focus the two light beams into the pupil. For narrowband illumination, the light from laser diodes, with center wavelengths of 447 nm (LP450-SF15; Thorlabs) or 637 nm (LP637-SF50; Thorlabs), was coupled into the system. The laser diodes were controlled by a Thorlabs external driver unit (CLD 1010, a compact laser diode and temperature controller). This driver unit enabled control of the laser power to achieve the desired output power. For the 637-nm light, an additional Thorlabs neutral density filter (NE20A, 2.0 OD) was placed at the input to reduce the overall intensity of the light levels used. For broadband light (550 ± 150 nm), a supercontinuum laser light source (SuperK COMPACT; NKT Photonics, Birkerød, Denmark) was coupled into the setup. The power for each wavelength was measured at the input level and the pupil plane using a power meter (843-R/843-R-USB; Newport Corporation, Irvine, CA, USA) to ensure an equal retinal illuminance of 140 ± 4 Td for each condition. The resultant output power at the pupil was about 40 nW for long-wavelength light, 145 nW for short-wavelength light, and 15 nW for broadband light.

### Illumination Stimuli

The illumination stimuli appeared as circular interference gratings with specific wavelengths (short, 450 nm; long, 635 nm; broadband, 450–700 nm) and covered a retinal area of 12° (Fig. 1, left). To prevent adaptation to the stimuli, the orientation and bandwidth of the grating randomly alternated among 0°, 45°, 90°, and 180° and 3, 6, 9, 12, and 18 cycles per degree every 5 seconds.

### Experimental Procedure

To account for diurnal fluctuations in axial length and choroidal thickness, the study measurements were conducted between 8 AM and 12 PM.^19^ Additionally, to avoid the reduction of interaction effects that could happen with a short washout period and to maintain the comfort of the participants, the different illumination conditions were tested on separate days. Before each measurement, participants underwent a 10-minute washout period in a dark room without near tasks to avoid axial length confounding factors such as accommodation or exercise.^20^^,^^21^

After the washout period, baseline choroidal thickness covering a retinal area of 42° was obtained from the central retina using swept-source OCT (ZEISS PLEX Elite 9000; Carl Zeiss Meditec, Dublin, CA, USA). Tracking mode and follow-up mode were used to ensure the same scanning position for following scans. The axial length was also measured using swept-source biometry (ZEISS IOLMaster 700). The optical path length from the anterior surface of the cornea to the retinal pigment epithelium is measured as axial length by IOLMaster.^22^ For each eye, an automated series of five readings was obtained and subsequently averaged to derive a single axial length value. During the 30-minute stimulation phase of the dominant eye, the non-dominant eye was occluded with an eye patch and served as the control eye throughout the data analysis. To avoid any bias due to the order of the used light, a group of participants (*n* = 9) started with short wavelength light and then was illuminated with long wavelength light in their next appointment and vice versa. Broadband illumination, which was tested only in a subset (*n* = 13), was always last. Choroidal thickness and axial length were measured immediately, a few seconds after the illumination period, and always starting with the test eye. After the measurements, the participants were asked to remain seated in the darkened room for another 30 minutes. Thereafter, choroidal thickness and axial length were measured again to check for recovery processes. In summary, an individual appointment lasted a total of 90 minutes.

### Axial and Choroidal Data Analysis

**Figure 2.**
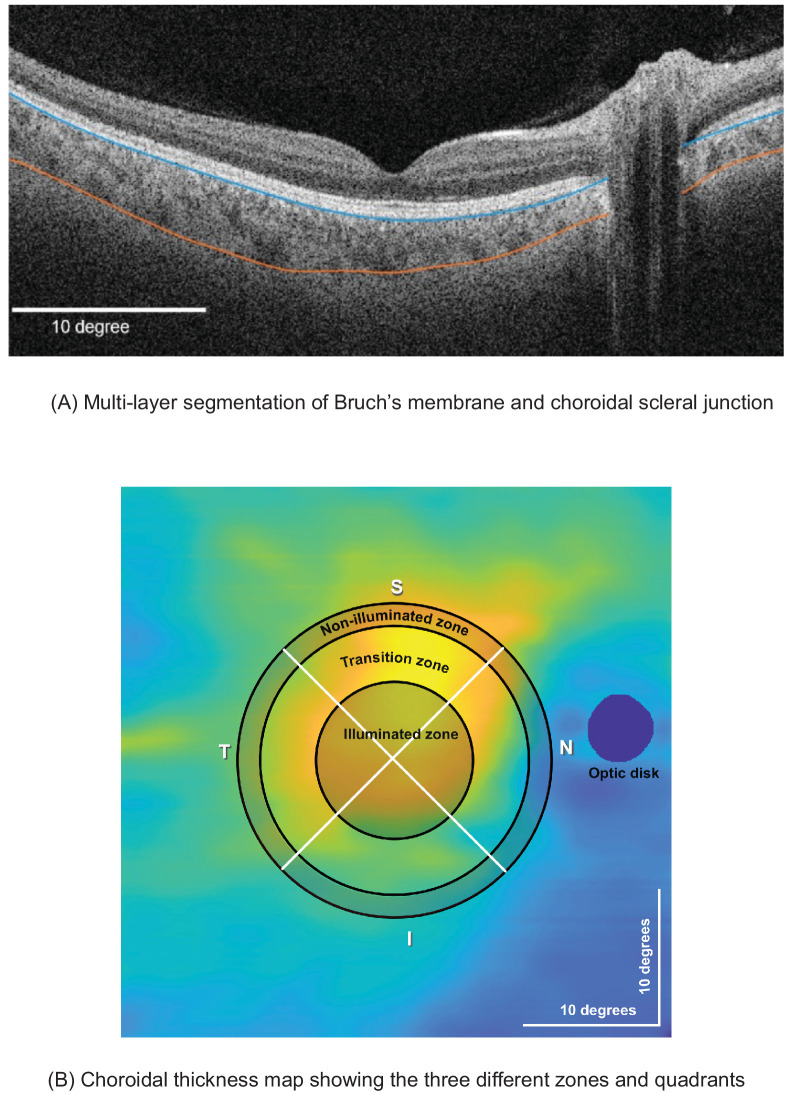
(A) Example segmentation of Bruch's membrane (*blue*) and choroidal–scleral junction (*orange*) of the left eye. (B) Choroidal thickness map of the right eye showing the illuminated transition non-illuminated zone and the four quadrants (S, superior; I, inferior; T, temporal; N, nasal).

The axial length data were directly obtained from the IOLMaster. For choroidal thickness, the 42° × 42° scans were uploaded onto the ZEISS Advanced Research and Imaging (ARI) Network, where an automated multilayer segmentation of Bruch's membrane and choroidoscleral junction was performed.^23^ The resulting segmentation results were then imported into MATLAB 2022b (MathWorks, Natick, MA, USA), where the segmentations of Bruch's membrane and the choroidal–scleral junction were carefully examined and manually adjusted if necessary (Fig. 2A). A two-dimensional 512 × 512 pixel choroidal thickness map (Fig. 2B) was obtained by taking the difference between Bruch's membrane and the choroidal–scleral junction for each A-scan and B-scan position. To derive the thickness of the choroid in micrometers, pixel values were converted to micrometers using a conversion factor of 1.9531.^24^ The baseline axial length and choroidal thickness measurement performed on each visit before the illumination phase were used to check for good repeatability of measurements assessed by an interclass correlation (ICC) analysis.

Assuming the fovea to be the center of the scan, the sub-retinal choroidal thickness was divided into three zones. First, the illumination zone: the area of illumination on the retina was 12° (Fig. 2B), and the full optical angle at the iris was 42°. Using these values, the resulting area of illumination in pixels was calculated to be 146 pixels in diameter from the center (fovea). Second, the outer non-illuminated zone (Fig. 2B): was calculated by extending an additional 146 pixels (parafoveal) in diameter from the illuminated zone. Third the transition zone: To have a completely non-illuminated zone, free from any influence of the illuminated stimuli, and also to maintain a nearly equal number of pixels in both the illuminated and non-illuminated zones, this transition zone in between was eliminated.

### Statistical Analysis

**Figure 3.**
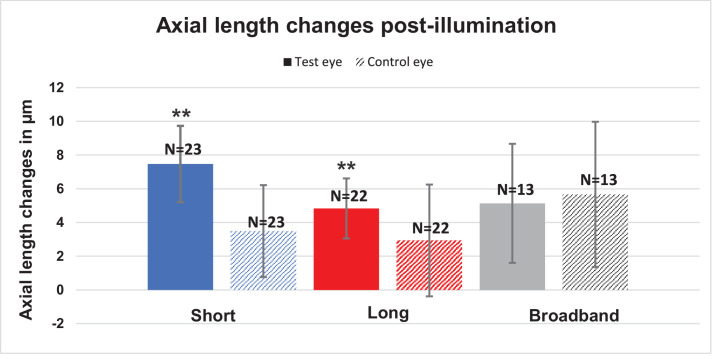
Mean axial length changes during the post-illumination period for the test eyes (*colored bars*) and control eyes (*patterned* *bars*). The asterisk (*) indicates significance.

**Figure 4.**
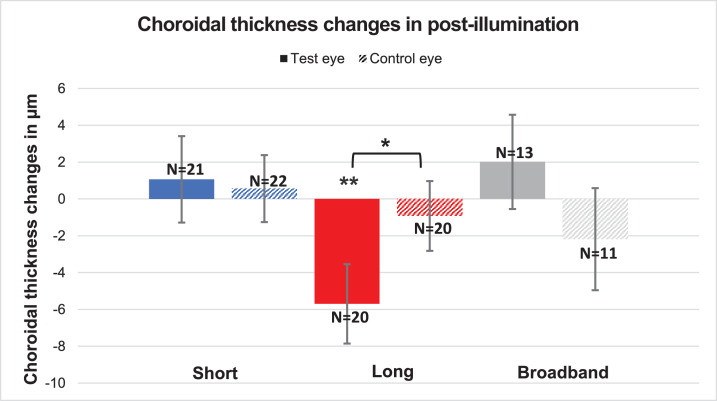
Mean choroidal thickness changes during the post-illumination period for the test eyes (*colored bars*) and control eyes (*patterned* *bars*). The asterisk (*) indicates significance.

**Figure 5.**
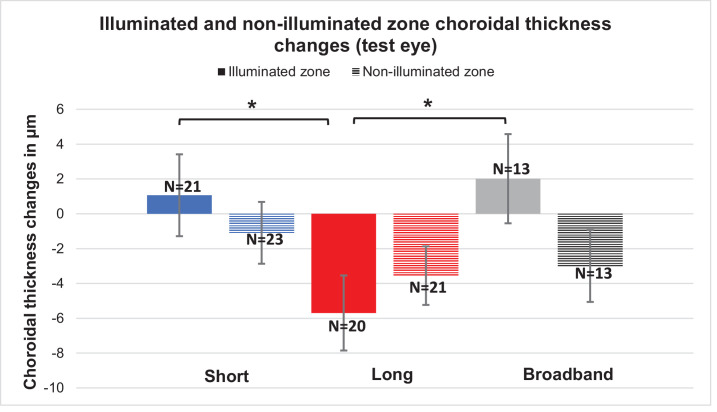
Mean illuminated zone (*colored bars*) and non-illuminated zone (*patterned* *bars*) choroidal thickness changes in test eyes post-illumination. The asterisk (*) indicates significance.

**Figure 6.**
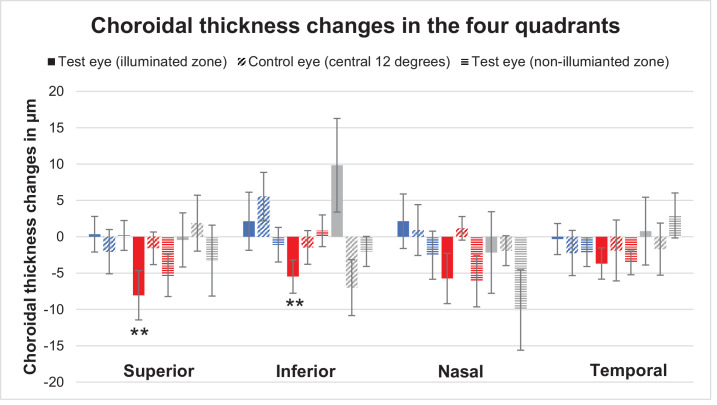
Mean choroidal thickness changes in superior, inferior, nasal and temporal quadrants for test eye illuminated zone (filled bars), control eye central 12 degrees (horizontal stripes) and non-illuminated zone of test eye (diagonal stripes). The asterisk (*) indicates significance.

**Figure 7.**
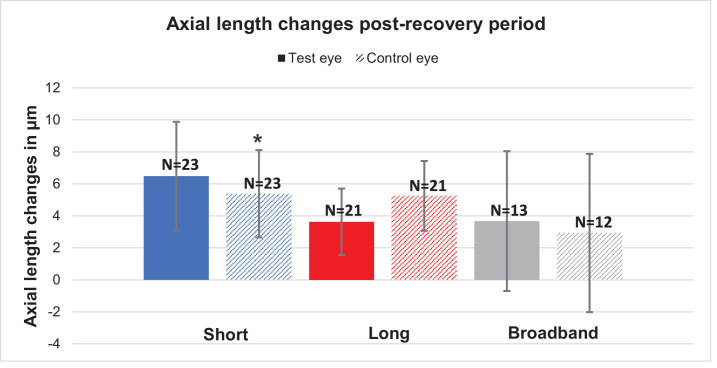
Mean axial length changes during the post-recovery period for the test eyes (*colored bars*) and control eyes (*patterned bars*). The asterisk (*) indicates significance.

Statistical analysis was performed using SPSS Statistics 29.0.0 (IBM, Chicago, IL, USA). Box plots were created to identify outliers, defined as data points significantly far from the center “box” based on 1.5 times the interquartile range of the upper and lower quartiles of the data.^25^ The outliers detected in this way were excluded from the following analysis. The number of participants excluded differed for each condition and eye; the final number of participants included in the analysis for each condition is stated in Figures 3 to 7. The normal distribution of post-illumination differences in axial length, choroidal thickness, and recovery measurement for both eyes was confirmed using the Shapiro–Wilk test. With the exception of the recovery measurement for axial length in the control eye for long-wavelength light, all data exhibited a normal distribution. The one-sample *t*-test was used to evaluate changes in axial length and choroidal thickness from baseline after the illumination period and after the 30-minute recovery period. A paired-samples test was carried out to determine if there was any significant difference between the test eye and the covered eye. Testing for significant differences among the illumination conditions is required to address the unequal participant distribution across conditions (450 nm, *n* = 23; 637 nm, *n* = 22; 550 ± 150 nm, *n* = 13). Thus, a linear mixed model with participants considered as a random factor and the condition as the fixed factor was used. Subsequently, Bonferroni correction was applied for multiple comparisons, facilitating examination of the relationships among the three illumination conditions and identifying any significant differences between the 12° illuminated zone and the non-illuminated zone.

## Results

### Post-Illumination Period Changes

Axial length and choroidal thickness changes after a 30-minute illumination period with short-wavelength, long-wavelength, and broadband light are shown in Figures 3 and 4. The exact numbers of these and the following data plots are provided in the Supplementary Material. An increase in axial length from baseline was noted in the post-illumination period with short-wavelength (7.4 ± 2.2 µm; *P* < 0.01) and long-wavelength (4.8 ± 1.7 µm; *P* = 0.01) light. After the broadband light stimulation, the eyes also showed an increase in axial length but without any significance (5.1 ± 3.5 µm; *P* = 0.08). The fellow eye, used as control, showed a non-significant increase in axial length, as well, across all conditions (all *P* > 0.05).

A significant thinning of the choroid was observed after long-wavelength light illumination (−5.7 ± 2.2 µm; *P* = 0.01). However, the other two conditions—short-wavelength light and broadband light—showed no significance (1.1 ± 2.3 µm and 2.0 ± 2.6 µm, respectively; both *P* > 0.05). Similarly, the control eye also showed no significant difference (all *P* > 0.05).

Comparing the change between the test and control eye, no significant difference was found for any of the light conditions. Only for long-wavelength light was a significant difference noted in choroidal thickness between the test and control eye (–5.7 ± 2.2 and –0.9 ± 1.9 µm, respectively; *P* = 0.04). A comparison of the light conditions also showed no significant changes in the test eye (short-wavelength vs. long-wavelength vs. broadband, all *P* > 0.05).

For a more detailed analysis, the actual stimulated area on the retina (illuminated zone) was compared against the periphery (non-illuminated zone). The illuminated zone choroidal thicknesses did not differ significantly from the non-illuminated within the conditions (all *P* > 0.05, Fig. 5). In contrast, the illuminated zones after stimulation with long-wavelength light showed significant differences in choroidal thickness compared to short wavelength light (*P* = 0.02) as well as broadband light (*P* = 0.02).

Additionally, for an even more detailed analysis, the retina was sectioned into four quadrants: superior, Inferior, nasal, and temporal (Fig. 6). No significant changes from baseline were observed for short and broadband wavelength lights for the test and control eye (all, *P* > 0.05). For long-wavelength light, in most quadrants and conditions the changes from baseline were non-significant, too (*P* > 0.05). Only within the test eye's illumination zone a significant thinning from baseline was observed for the superior (*P* = 0.01) and inferior (*P* = 0.01) quadrants and the control eye showed no significance (all, *P* > 0.05).

### Changes After the Recovery Period

**Figure 8.**
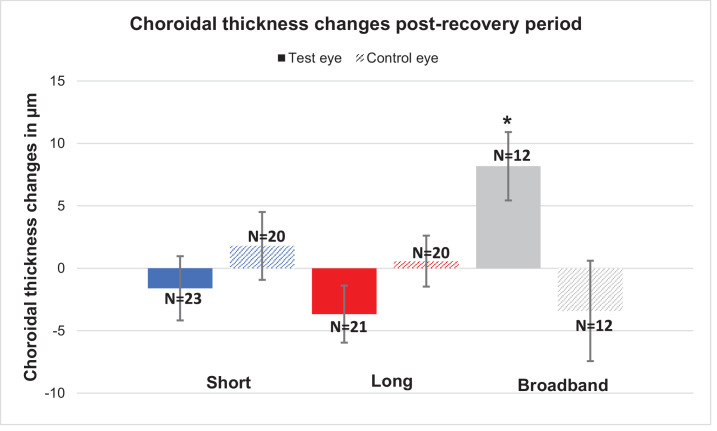
Mean choroidal thickness changes during the post-recovery period for the test eyes (*colored bars*) and control eyes (*patterned bars*). The asterisk (*) indicates significance.

Axial length and choroidal thickness changes after a 30-minute recovery period with short-wavelength, long-wavelength, and broadband light are shown in Figures 7 and 8. The axial length showed no significant change for any of the conditions in the test eye after a 30-minute recovery period (all *P* > 0.05). The control eye showed a significant difference in the case of short-wavelength light (6.1 ± 2.8 µm; *P* = 0.04), and the other two light conditions of long-wavelength and broadband light showed no significant difference. The choroidal thickness showed a significant difference after the recovery period for broadband light in the test eye (8.2 ± 2.7 µm; *P* = 0.01). However, the short-wavelength (*P* = 0.53) and long-wavelength (*P* = 0.12) light showed no significant differences, and the covered eyes also showed no significant differences for any of the three conditions (all *P* > 0.05).

## Discussion

Building on the pilot study of Breher et al.,^16^ who investigated changes in axial length in response to aberration-free narrowband light, the current study used higher light intensities (2.15 logTd instead of 0.6 logTd) for a longer stimulation time (30 minutes instead of 20 minutes). The results observed in this study suggest that a 30-minute exposure to different light wavelengths in the absence of defocus induced by LCA induces eye changes that are not sufficient to regulate eye growth.

Different results were observed compared to the previous study by Breher et al.^16^ The previous study reported no significant change for any of the conditions, whereas in the current study there was a significant change in axial length in the case of short- and long-wavelength light, presumably due to the significantly higher light intensities and longer stimulation period used here. However, axial length significantly increased for both illumination scenarios, which seems to be contradictory to the observations from other studies where the LCA was not controlled for. Thakur et al.^11^ found that axial length decreased in response to short-wavelength light (−8.0 ± 2.7 µm) and increased in response to long-wavelength light (11.2 ± 2.0 µm). Similarly,^10^ noted a significant increase in axial length for long-wavelength light and additionally reported a small but non-significant increase for short-wavelength light, suggesting that the eye was triggered by the defocus due to LCA, thus supporting the notion that the wavelength acted as a target. In combination with the findings reported here, it could be suggested that LCA-induced defocus plays a greater role compared with chromaticity in triggering eye changes.

In the current study, a decrease in choroidal thickness for long-wavelength light was noted, which is consistent with the results of Lou and Ostrin,^10^ as well as those of Thakur et al.^11^ However, no significant change was noted in the case of short-wavelength light. Previous studies used natural viewing conditions and commercial lighting systems, which do not control for LCA induced defocus. The setup used in this study provides an aberration free stimulation without any LCA, which leads to the conclusion that the observed choroidal thinning is rather triggered by another mechanism other than defocus. Although the duration of illumination was longer compared to the previous studies by Breher et al.,^16^ 30 minutes is still a relatively short duration compared to previous studies where the eye was illuminated for an hour.^10^^,^^11^ Additionally, accommodation is thought to lead to an increase in axial length and thinning of choroidal thickness,^20^ but accommodation, which could have been triggered due to dust in the optics, was not controlled for in our study.

Recently, treatment with low-level red-light therapy to control myopia is gaining more attention because it has proven to be effective in controlling myopia progression.^12^^,^^13^^,^^26^ The exact mechanism of defocus caused by LCA is not clear in these studies. Gawne et al.^27^ proposed that an imbalance between short-wavelength sensitive (SWS) and long-wavelength sensitive (LWS) opsin drives the eye in the direction where both are balanced. For example, under red light illumination, the LWS is overactivated and SWS is not activated, which in turn leads the eye to develop in the direction of hyperopia.^27^ This was not true in our study, because illumination with long-wavelength light resulted in axial elongation and choroidal thinning.

No significant differences were observed when comparing changes in axial length and choroidal thickness between the illumination conditions. These similar changes in axial length could be due to factors such as increased light intensity of the stimuli^28^ or the influence of diurnal rhythm, which was also noted in another study, where the axial length was found to be the longest around noon.^29^ However, the changes that happened during the illumination period returned to their initial stage after a 30-minute recovery period, indicating that the diurnal eye rhythm was not the main trigger.

Axial length showed good repeatability with an interclass correlation (ICC) greater than 0.9, which is similar to other results with the IOLMaster 700 which showed ICC values greater than 0.9.^30^^,^^31^ Previous studies have also reported an ICC value greater than 0.8 for choroidal thickness.^32^^,^^33^ Similarly, in our study, choroidal thickness showed an ICC greater than 0.9, indicating good repeatability.

In the present study, the eye showed no significant change in reaction to the control condition or broadband light. However, there was a trend for the axial length to increase (5.1 ± 3.5 µm), again suggesting that the eye reacts to increased intensity of stimuli. Choroidal thickness also showed no significant change in reaction to broadband light, but a slight tendency for the thickness to increase was noticed (2.0 ± 2.6 µm). Moreover, it should also be noted that the lack of significant results can be due to the low sample size of 13 participants.

The control eye showed a non-significant tendency to increase in axial length similar to the test eye for all wavelengths. Additionally, the choroidal thickness in the control eye responded similarly to the different wavelengths of light. When comparing the interactions between the test and control eyes, no significance was found, indicating that both eyes responded similarly to all of the wavelength conditions. It can be hypothesized that the non-significant changes observed in the fellow eye may reflect residual diurnal variation or a response to darkness. Similar results were found in another study in which axial length increased when exposed to darkness.^10^ Thus, the observed changes in the other eye could be due to external factors rather than a direct response to the differences in the light spectrum. This is then also true for the test eye, as only the central 12° of the retina was illuminated and the peripheral retina was dark, which could have also triggered the thinning of the choroid or elongation of axial length.

The non-illuminated versus illuminated zone showed no significant difference in choroidal thickness within and between the conditions, indicating that any change (e.g., significant choroidal thinning with long-wavelength illumination) is limited not only to the illuminated zone but also to the surrounding non-illuminated area. Previous studies reported that, in myopia, the choroid thinned more in the foveal region than in the outer macular region, a difference that could indicate more myopiagenic activity in the central retina compared to the peripheral retina.^34^^,^^35^ In our study, this inconsistency could be also due to the used stimulus size, as only the central retinal region was illuminated.

## Conclusions

In conclusion, axial length reacted similarly for short-wavelength, long-wavelength, and broadband light conditions. Also, choroidal thickness decreased in the case of long-wavelength light. Therefore, light without associated defocus induced by LCA seems to be insufficient to regulate eye growth in a chromaticity-specific manner.

## Supplementary Material

Supplement 1
