# Supplementary material for: Influence of Aberration-Free, Narrowband Light on the Choroidal Thickness and Eye Length
Source: Transl Vis Sci Technol. 2024 Apr 25;13(4):30. doi: 10.1167/tvst.13.4.30 (PMC11055502; doi:10.1167/tvst.13.4.30)
Supplement: Supplement 1 [file tvst-13-4-30_s001.pdf]

Supplementary Tables

Table 1: Axial length and choroidal thickness changes post-illumination and post-recovery period for test and control eye and choroidal thickness changes of the illuminated and non-illuminated zone for the test eye post-illumination period. Results are shown in micrometer and mean ± standard error.

**Post-illumination period**				
	**Axial length**		**Choroidal thickness**	
**Illumination conditions**	**Test eye**	**Control eye**	**Test eye**	**Control eye**
**Short wavelength**	+7.4 ± 2.2	+3.5 ± 2.7	+1.1 ± 2.3	+0.6 ± 1.8
**Long wavelength**	+4.8 ± 1.7	+2.9 ± 3.3	-5.7 ± 2.2	-0.9 ± 1.9
**Broadband**	+5.1 ± 3.5	+5.6 ± 4.3	+2.0 ± 2.6	-2.2 ± 2.8
**Post-recovery period**				
	**Axial length**		**Choroidal thickness**	
**Illumination conditions**	**Test eye**	**Control eye**	**Test eye**	**Control eye**
**Short wavelength**	+6.5 ± 3.3	+5.4 ± 2.7	-1.6 ± 2.3	+1.8 ± 2.7
**Long wavelength**	+3.6 ± 2.1	+5.2 ± 2.1	-3.7 ± 2.3	+0.6 ± 2
**Broadband**	+3.6 ± 4.3	+2.9 ± 4.9	+8.2 ± 2.7	-3.4 ± 4

**Illuminated and non-illuminated zone choroidal thickness**		
	**Test eye (post-illumination period)**	
**Illumination conditions**	**Illuminated zone**	**Non-illuminated zone**
**Short wavelength**	+1.1 ± 2.3	-1.1 ± 1.8
**Long wavelength**	-5.7 ± 2.2	-3.5 ± 1.7
**Broadband**	+2.0 ± 2.6	-3.0 ± 2.1

Table 2: choroidal thickness changes post-illumination period for test eye in four quadrants of illuminated and non-illuminated zone.

Choroidal thickness changes post-illumination (test eye)					
		**Superior**	**Inferior**	**Nasal**	**Temporal**
**Short wavelength**	**illuminated**	+0.3 ± 2.4	+2.1 ± 4.0	+2.1 ± 3.8	-0.3 ± 2.1
	**Non-illuminated**	+0.2 ± 2.1	-1.1 ± 2.4	-2.5 ± 3.3	-2.1 ± 2.0
**Long wavelength**	**illuminated**	-8.1 ± 3.4	-5.5 ± 2.1	-5.7 ± 3.5	-3.7 ± 2.2
	**Non-illuminated**	-5.3 ± 2.9	+0.8 ± 2.1	-6.1 ± 3.5	-3.6 ± 1.6
**Broadband**	**illuminated**	-0.4 ± 3.7	+9.8 ± 6.4	-2.2 ± 5.6	+0.7 ± 4.7
	**Non-illuminated**	-3.3 ± 4.9	-2.0 ± 2.1	-10.0 ± 5.5	+2.9 ± 3.1

Table 3: choroidal thickness changes post-illumination period for the control eye in four quadrants of the central 12-degree zone.

Choroidal thickness changes in the central zone post-illumination (control eye)				
	**Central 12 degrees**			
	**Superior**	**Inferior**	**Nasal**	**Temporal**
**Short wavelength**	-2.0 ± 3.0	+5.5 ± 3.3	+0.9 ± 3.1	-2.2 ± 3.5
**Long wavelength**	-1.6 ± 2.2	-1.5 ± 2.3	+1.2 ± 1.6	-1.9 ± 4.2
**Broadband**	+1.8 ± 3.8	-7.0 ± 3.8	-1.9 ± 3.6	-1.7 ± 2.1

On analysing the four different quadrants, no significant difference was observed for any of the conditions (all $p > 0.05$).
